# 4-Chloro-2,6-dinitro­phenol

**DOI:** 10.1107/S1600536810046490

**Published:** 2010-11-17

**Authors:** Seik Weng Ng

**Affiliations:** a Department of Chemistry, University of Malaya, 50603 Kuala Lumpur, Malaysia

## Abstract

The aromatic ring of the title compound, C_6_H_3_ClN_2_O_5_, is almost planar (r.m.s. deviation = 0.007 Å); one nitro substituent is nearly coplanar with the ring [dihedral angle = 3(1)°], whereas the other is twisted [dihedral angle = 36 (1)°]. The phenol OH group is intra­molecularly hydrogen bonded to the nitro group that is coplanar with the ring, generating an *S*(6) graph-set motif.

## Related literature

For the crystal structure of picric acid, see: Duesler *et al.* (1978 ▶); Soriano-Garcia *et al.* (1980 ▶).
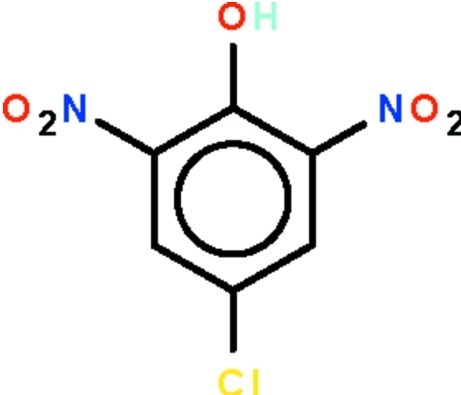


## Experimental

### 

#### Crystal data

C_6_H_3_ClN_2_O_5_
*M* *_r_* = 218.55
Monoclinic, 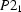

*a* = 7.4700 (19) Å
*b* = 5.8973 (15) Å
*c* = 9.952 (2) Å
β = 109.939 (6)°
*V* = 412.13 (18) Å^3^
*Z* = 2
Mo *K*α radiation
μ = 0.46 mm^−1^
*T* = 293 K
0.24 × 0.21 × 0.18 mm

#### Data collection

Rigaku R-AXIS RAPID diffractometer
Absorption correction: multi-scan (*ABSCOR*; Higashi, 1995 ▶) *T*_min_ = 0.897, *T*_max_ = 0.922
3209 measured reflections
1434 independent reflections
816 reflections with *I* > 2σ(*I*)
*R*_int_ = 0.067

#### Refinement

*R*[*F* ^2^ > 2σ(*F* ^2^)] = 0.051
*wR*(*F* ^2^) = 0.098
*S* = 1.01
1434 reflections
131 parameters
2 restraints
H atoms treated by a mixture of independent and constrained refinement
Δρ_max_ = 0.24 e Å^−3^
Δρ_min_ = −0.24 e Å^−3^
Absolute structure: Flack (1983 ▶), 640 Friedel pairs
Flack parameter: 0.14 (14)

### 

Data collection: *RAPID-AUTO* (Rigaku, 1998 ▶); cell refinement: *RAPID-AUTO*; data reduction: *CrystalStructure* (Rigaku/MSC, 2002 ▶); program(s) used to solve structure: *SHELXS97* (Sheldrick, 2008 ▶); program(s) used to refine structure: *SHELXL97* (Sheldrick, 2008 ▶); molecular graphics: *X-SEED* (Barbour, 2001 ▶); software used to prepare material for publication: *publCIF* (Westrip, 2010 ▶).

## Supplementary Material

Crystal structure: contains datablocks global, I. DOI: 10.1107/S1600536810046490/hg2748sup1.cif

Structure factors: contains datablocks I. DOI: 10.1107/S1600536810046490/hg2748Isup2.hkl

Additional supplementary materials:  crystallographic information; 3D view; checkCIF report

## Figures and Tables

**Table 1:** Hydrogen-bond geometry (Å, °)

*D*—H⋯*A*	*D*—H	H⋯*A*	*D*⋯*A*	*D*—H⋯*A*
O3—H3⋯O4	0.84 (6)	1.82 (4)	2.563 (6)	146 (7)

**Fig. 1.**
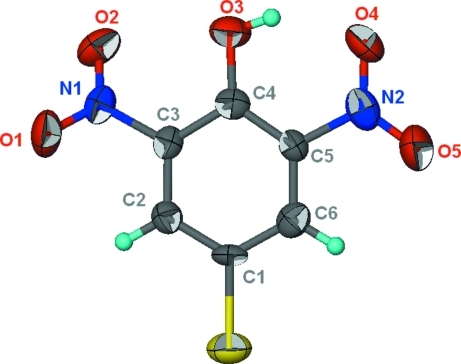
Thermal ellipsoid plot (Barbour, 2001) of 4-chloro-2,6-dinitrophenol at the 50% probability level; hydrogen atoms are drawn as spheres of arbitrary radius.

## supplementary crystallographic information

### Comment

2,4,6-Trinitrophenol (picric acid) is a strong oxygen acid that dissociates in water. In the solid state, the molecule is nearly flat (Duesler *et al.*, 1978; Soriano-Garcia *et al.*, 1980). 4-Chloro-2,6-dinitrophenol (Scheme I) is also a similarly strong oxygen acid as it dissociates in water completely in water. In the crystal structure, the aromatic ring is nearly co-planar with one nitro substituent (dihedral angle 3(1) °) whereas it is twisted with respect to the other (dihedral angle 36 (1) °) (Fig. 1). The phenolic group is intra-molecularly hydrogen bonded to the nitro group that is co-planar with the ring.

### Experimental

Commercially available 4-chloro-2,6-dinitrophenol was recrystallized from methanol to yield colorless prisms.

### Refinement

Hydrogen atoms were placed in calculated positions (C–H 0.93 Å) and were included in the refinement in the riding model approximation, with *U*(H) set to 1.2*U*_eq_(C). The hydroxy H-atom was located in a difference Fourier map, and was refined with a distance restraint of O–H 0.84±0.01 Å.

### Crystal data

**Table:**

C_6_H_3_ClN_2_O_5_	*F*(000) = 220
*M**_r_* = 218.55	*D*_x_ = 1.761 Mg m^−^^3^
Monoclinic, *P*2_1_	Mo *K*α radiation, λ = 0.71073 Å
Hall symbol: P 2yb	Cell parameters from 1662 reflections
*a* = 7.4700 (19) Å	θ = 4.1–27.4°
*b* = 5.8973 (15) Å	µ = 0.46 mm^−^^1^
*c* = 9.952 (2) Å	*T* = 293 K
β = 109.939 (6)°	Prism, colorless
*V* = 412.13 (18) Å^3^	0.24 × 0.21 × 0.18 mm
*Z* = 2	

### Data collection

**Table:**

Rigaku R-AXIS RAPID diffractometer	1434 independent reflections
Radiation source: fine-focus sealed tube	816 reflections with *I* > 2σ(*I*)
graphite	*R*_int_ = 0.067
Detector resolution: 10.000 pixels mm^-1^	θ_max_ = 25.0°, θ_min_ = 4.1°
ω scans	*h* = −8→8
Absorption correction: multi-scan (*ABSCOR*; Higashi, 1995)	*k* = −7→7
*T*_min_ = 0.897, *T*_max_ = 0.922	*l* = −11→11
3209 measured reflections	

### Refinement

**Table:**

Refinement on *F*^2^	Secondary atom site location: difference Fourier map
Least-squares matrix: full	Hydrogen site location: inferred from neighbouring sites
*R*[*F*^2^ > 2σ(*F*^2^)] = 0.051	H atoms treated by a mixture of independent and constrained refinement
*wR*(*F*^2^) = 0.098	*w* = 1/[σ^2^(*F*_o_^2^) + (0.0358*P*)^2^] where *P* = (*F*_o_^2^ + 2*F*_c_^2^)/3
*S* = 1.01	(Δ/σ)_max_ = 0.001
1434 reflections	Δρ_max_ = 0.24 e Å^−^^3^
131 parameters	Δρ_min_ = −0.24 e Å^−^^3^
2 restraints	Absolute structure: Flack (1983), 640 Friedel pairs
Primary atom site location: structure-invariant direct methods	Flack parameter: 0.14 (14)

### Fractional atomic coordinates and isotropic or equivalent isotropic displacement parameters (Å^2^)

**Table:**

	*x*	*y*	*z*	*U*_iso_*/*U*_eq_	
Cl1	0.4887 (2)	0.0000 (3)	0.86383 (14)	0.0688 (5)	
O1	0.3354 (6)	0.5743 (6)	0.4395 (4)	0.0733 (13)	
O2	0.2527 (5)	0.8807 (6)	0.5218 (4)	0.0721 (13)	
O3	−0.0108 (6)	0.7765 (6)	0.6318 (4)	0.0604 (12)	
H3	−0.101 (7)	0.774 (12)	0.664 (7)	0.10 (3)*	
O4	−0.1986 (6)	0.6424 (8)	0.7898 (4)	0.0729 (14)	
O5	−0.1056 (7)	0.3531 (8)	0.9292 (5)	0.0845 (14)	
N1	0.2807 (6)	0.6773 (8)	0.5258 (5)	0.0497 (12)	
N2	−0.0885 (7)	0.4861 (11)	0.8388 (5)	0.0594 (13)	
C1	0.3379 (8)	0.2247 (7)	0.7937 (5)	0.0383 (13)	
C2	0.3679 (7)	0.3605 (9)	0.6918 (6)	0.0430 (13)	
H2	0.4672	0.3293	0.6582	0.052*	
C3	0.2500 (6)	0.5442 (8)	0.6390 (5)	0.0376 (13)	
C4	0.0974 (7)	0.5968 (8)	0.6866 (6)	0.0425 (13)	
C5	0.0733 (7)	0.4491 (8)	0.7875 (5)	0.0387 (14)	
C6	0.1891 (8)	0.2652 (9)	0.8411 (6)	0.0479 (15)	
H6	0.1666	0.1703	0.9083	0.057*	

### Atomic displacement parameters (Å^2^)

**Table:**

	*U*^11^	*U*^22^	*U*^33^	*U*^12^	*U*^13^	*U*^23^
Cl1	0.0706 (9)	0.0606 (9)	0.0678 (10)	0.0225 (9)	0.0142 (8)	0.0110 (9)
O1	0.098 (3)	0.069 (3)	0.075 (3)	−0.012 (2)	0.058 (3)	0.002 (2)
O2	0.090 (3)	0.050 (3)	0.075 (3)	−0.002 (2)	0.027 (3)	0.018 (2)
O3	0.055 (3)	0.048 (2)	0.077 (3)	0.011 (2)	0.021 (3)	0.008 (2)
O4	0.063 (3)	0.083 (3)	0.084 (3)	0.023 (3)	0.038 (3)	0.001 (3)
O5	0.093 (3)	0.093 (3)	0.092 (4)	0.009 (3)	0.062 (3)	0.012 (3)
N1	0.052 (3)	0.055 (3)	0.043 (3)	−0.011 (2)	0.017 (3)	0.003 (3)
N2	0.062 (4)	0.065 (3)	0.057 (3)	−0.005 (3)	0.028 (3)	−0.019 (3)
C1	0.037 (3)	0.027 (3)	0.044 (3)	0.011 (2)	0.005 (3)	0.003 (2)
C2	0.039 (3)	0.047 (3)	0.044 (3)	0.000 (3)	0.016 (3)	−0.001 (3)
C3	0.034 (3)	0.042 (4)	0.034 (3)	−0.009 (3)	0.009 (2)	−0.002 (2)
C4	0.037 (3)	0.042 (3)	0.044 (3)	−0.001 (3)	0.007 (3)	−0.003 (3)
C5	0.037 (3)	0.041 (4)	0.040 (3)	−0.002 (3)	0.016 (3)	−0.005 (3)
C6	0.055 (4)	0.048 (3)	0.037 (3)	−0.004 (3)	0.012 (3)	−0.002 (3)

### Geometric parameters (Å, °)

**Table:**

Cl1—C1	1.725 (4)	C1—C6	1.369 (7)
O1—N1	1.230 (5)	C1—C2	1.369 (6)
O2—N1	1.216 (5)	C2—C3	1.382 (7)
O3—C4	1.332 (6)	C2—H2	0.9300
O3—H3	0.84 (6)	C3—C4	1.410 (6)
O4—N2	1.221 (6)	C4—C5	1.387 (6)
O5—N2	1.233 (6)	C5—C6	1.376 (7)
N1—C3	1.454 (6)	C6—H6	0.9300
N2—C5	1.480 (6)		
			
C4—O3—H3	107 (5)	C2—C3—C4	121.9 (5)
O2—N1—O1	124.0 (5)	C2—C3—N1	118.0 (4)
O2—N1—C3	119.2 (5)	C4—C3—N1	120.1 (5)
O1—N1—C3	116.9 (5)	O3—C4—C5	125.9 (5)
O4—N2—O5	123.3 (5)	O3—C4—C3	119.0 (5)
O4—N2—C5	119.5 (5)	C5—C4—C3	115.1 (4)
O5—N2—C5	117.3 (6)	C6—C5—C4	123.8 (4)
C6—C1—C2	120.7 (5)	C6—C5—N2	117.5 (5)
C6—C1—Cl1	119.3 (4)	C4—C5—N2	118.7 (5)
C2—C1—Cl1	120.0 (4)	C1—C6—C5	118.7 (5)
C1—C2—C3	119.7 (4)	C1—C6—H6	120.6
C1—C2—H2	120.1	C5—C6—H6	120.6
C3—C2—H2	120.1		

### Hydrogen-bond geometry (Å, °)

**Table:**

*D*—H···*A*	*D*—H	H···*A*	*D*···*A*	*D*—H···*A*
O3—H3···O4	0.84 (6)	1.82 (4)	2.563 (6)	146 (7)
